# Comparison of Emergence Agitation Scale Scores and Creatine Kinase Levels After General Anesthesia in Children: A Prospective Cohort Study

**DOI:** 10.7759/cureus.26991

**Published:** 2022-07-18

**Authors:** Mayumi Hashimoto, Aiji Sato-Boku, Naoko Tachi, Yoko Okumura, Masahiro Okuda, Yoko Goto, HIdekazu Ito, Toshiyuki Kitoh

**Affiliations:** 1 Department of Anesthesiology, Aichi Gakuin University, Nagoya, JPN; 2 Department of Pediatrics, Aichi Gakuin University Dental Hospital, Nagoya, JPN; 3 Department of Anesthesiology, Toyokawa City Hospital, Toyokawa, JPN; 4 Laboratory of Pediatrics, Aichi Gakuin University School of Pharmacy, Nagoya, JPN

**Keywords:** emergence agitation, malignant hyperthermia, muscle damage, creatine kinase, general anesthesia

## Abstract

Introduction

A simple indicator of muscle damage is creatine kinase (CK). Although CK elevation is informative for malignant hyperthermia, no study has examined the relationship between the anesthetically awake state and CK in children. We aimed to prospectively examine the relationship between the awakening state and CK on the day after surgery in children who have undergone anesthesia with volatile inhalation anesthetics.

Methods

The study included 119 patients aged 0-15 years and scheduled to undergo general anesthesia for cleft lip and palate-related surgery. Emergence agitation (EA) was assessed after completion of general anesthesia using the five-point EA scale, and patients were divided into the following five groups according to the EA score: EA1, EA2, EA3, EA4, and EA5. The primary outcome was ΔCK (comparison of CK values one week prior to surgery to CK values on the day after surgery) in each EA group. The secondary outcome was ΔCK when the EA score was divided into the following two groups: EA ≤2 (EA score of 1 or 2) and EA ≥3 (EA score of 3, 4, or 5).

Results

The median ΔCK values in the EA1 to EA5 groups were 3 (quartile −19~9), 5 (−32~88), 99.5 (−18~190.5), 121 (29~219.5), and 144 (41~340.5), respectively, indicating a statistically significant difference overall. Statistically significant differences were also observed between the EA1 and EA4 groups and between the EA2 and EA4 groups. The median ΔCK values in the EA ≤2 and EA ≥3 groups were 3 (quartile −27~85) and 108 (23.5~206.7), respectively, indicating a statistically significant difference.

Conclusion

The results of this study revealed that a higher EA score at the time of anesthesia awakening is associated with a larger ΔCK, indicating that a high CK level on the day after surgery is highly related to the state of the patient upon awakening.

## Introduction

A simple indicator of muscle damage is creatine kinase (CK), an enzyme found in large amounts in muscles. It plays an important role in muscle cell energy metabolism, and CK levels in the blood are high when muscles are damaged or destroyed. In the past, general anesthesia with volatile inhalation anesthetics has been shown to impair the stability of skeletal muscle cell membranes, with a higher degree of impairment in children [1].

One of the most serious complications in anesthesiology is malignant hyperthermia (MH). Malignant hyperthermia is induced by all volatile inhalant anesthetics and depolarizing muscle relaxants, such as succinylcholine [2,3], which increase the rate of calcium release from the sarcoplasmic reticulum [4,5], resulting in abnormal and sustained muscle contraction in skeletal muscles and subsequently causing the destruction of skeletal muscles and the production of large amounts of heat [6,7].

Although CK elevation is informative for MH diagnosis [8], strenuous movement, which occurs with awakening from pediatric general anesthesia, can cause it, and to the best of our knowledge, no study has examined the relationship between the anesthetically awake state and CK in children. Therefore, in this study, we decided to prospectively examine the relationship between the awakening state and CK on the day after surgery in children who have undergone anesthesia with volatile inhalation anesthetics, with consideration of age.

## Materials and methods

Ethics approval and consent to participate

This study was conducted in accordance with the ethical standards of the Declaration of Helsinki (1964) and its subsequent amendments. Moreover, this study was approved by the Ethics Committee at the School of Dentistry, Aichi Gakuin University (approval no.: 530). Written informed consent was obtained from the parents of all patients participating in the trial, and the protection of personal information was sufficiently considered.

Study design and population

We conducted a prospective cohort study in 123 patients aged 0-15 years and scheduled to undergo general anesthesia for cleft lip and palate-related surgery from May 2018 to April 2022 (single-center, academic). The exclusion criteria were the presence of muscle diseases, such as myasthenia gravis (n=0) and no consent (n=4). Therefore, the final study population included 119 patients. Emergence agitation (EA) was assessed after completion of general anesthesia using the five-point EA scale (1: sleeping; 2: awake, calm; 3: irritable, crying; 4: inconsolable, crying; 5: severe restlessness, disorientation) [9], and the patients were divided into the following five groups according to the EA score: EA1, EA2, EA3, EA4, and EA5. 

Anesthesia methods

The same method of anesthesia was employed for all patients. The standard vital sign measures (electrocardiogram, blood pressure, and oxygen saturation) were inspected. Anesthesia was induced by 5% sevoflurane. After the patient fell asleep, we placed an intravenous line, and endotracheal intubation was facilitated with 0.6 mg/kg rocuronium and fentanyl (1 μg/kg). Anesthesia was maintained with 2% end-tidal sevoflurane, 60% air in oxygen, and remifentanil (0.1-0.2 μg/kg/min). A local anesthetic (2% lidocaine containing adrenaline) was also injected into the operative site, and 10 minutes before the end of the surgery, fentanyl (1 μg/kg) was administered as a bolus to patients. At the end of the operation, anesthetic gases were discontinued, and patients received rectal acetaminophen (20 mg/kg). Extubation was performed upon the patient awakening. EA scores of the patients were evaluated after extubation by the anesthesiologist in charge. Patients were then transferred to the postanesthetic care unit (PACU). In the PACU, parents were allowed to be with their children. Supplemental oxygen was administered when SpO2 decreased to less than 95%. 

Measurements

The primary outcome was ΔCK (comparison of CK values one week prior to surgery to CK values on the day after surgery) in each EA group. The secondary outcome was ΔCK when the EA score was divided into the following two groups: EA ≤2 (EA score of 1 or 2) and EA ≥3 (EA score of 3, 4, or 5). In addition, receiver-operating curves (ROCs) curves were evaluated using ΔCK to predict EA. Patient background, operative time, anesthesia time, and operative details were also evaluated.

Statistical analysis

In statistical analyses, the chi-square test was used to assess the relationship between two categorical variables, the Mann-Whitney U test was used to compare differences between two independent groups for continuous variables, and the Kruskal-Wallis test was used to compare differences between three or more independent groups. The Steel-Dwass test was used for multiple comparisons. The two-sided statistical significance level was set at p≤0.05. To compare the power of ΔCK to predict EA, ROCs were plotted, and area under the curves (AUCs) were calculated. The optimal cut-off points were determined using ROC curves to maximize the sensitivity and specificity. Statistical analysis of recorded data was performed using SPSS version 26 (IBM Inc., Armonk, New York).

## Results

Table 1 presents the demographic characteristics, operation time, and anesthesia time. Of the 119 patients, 21 were included in the EA1 group, 44 were included in the EA2 group, 27 were included in the EA3 group, 19 were included in the EA4 group, and eight were included in the EA5 group. Statistically significant differences in patient age, height, and weight were observed between the groups. However, there were no significant differences in operation time and anesthesia time between the groups.

**Table 1:** Patients' demographic and related information Values are number or median (quartile 1-quartile 3)

		EA1		EA2		EA3		EA4		EA5		p-value			
																Male/female		16/5		24/20		9/18		14/5		7/1		p<0.01			
Age (month)		104 (89-123)		109 (74.75-120)		53 (17.5-118)		21 (12-27)		102 (93-109.25)		EA1 vs EA4, EA2 vs EA4, EA4 vs EA5 (p<0.01)																			
																Height (cm)		131.3 (122-136.9)		130.25 (111.35-136.75)		99.8 (77.9-136.4)		80 (73-84.05)		130.8 (125-134.5)		EA1 vs EA4, EA2 vs EA4, EA4 vs EA5 (p<0.01)			
Weight (kg)		28.45 (25-31)		25 (18.1875-30.125)		13.95 (9.8355-29.05)		10.53 (8.5475-11.815)		28.7 (25.775-31.025)		EA1 vs EA4, EA2 vs EA4, EA4 vs EA5 (p<0.01)																			
																Operation time (min)		100 (56-140)		115.5 (95.75-142.25)		115 (74-141)		118 (97-147)		66.5 (43.5-76.25)		NS			
Anesthesia time (min)		165 (101-208)		184.5 (145.75-209.25)		180 (144-226.5)		200 (152-229)		127.5 (83.75-139.75)		NS																			

Table 2 presents CK values and clinical data of pre- and postoperative patients. Pre and postoperative CK values tended to increase with decreasing age. The CK values of three cases out of 15 (20%) of less than one-year-old patients were above the age-adjusted normal limit. One case out of 57 (1.8%) of more than five-year-old and less than 10-year-old patients were above the age-adjusted normal limit. Three was no case in the other two age groups.

**Table 2:** CK values and clinical data of pre- and postoperative patients CK - creatine kinase Values are number or median (quartile 1-quartile 3)

Age (years)		<1		<5		<10		<15	
										Case		15		25		57		22	
Preoperative CK		157 (131-213)		103 (87-123)		112 (88-129)		112 (76-123)											
										Above normal limit case		3		0		1		0	
Above normal limit case (%)		20		0		1.8		0											
Postoperative CK		232 (169-301)		234 (121-353)		135 (92-229)		113 (64-207)	

**Figure 1:**
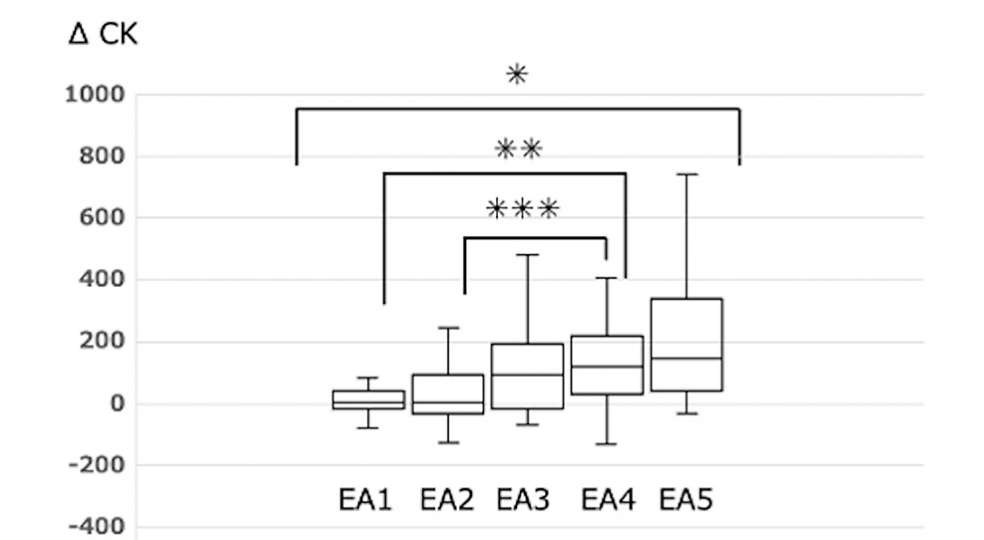
ΔCK in each EA group CK - creatine kinase; ΔCK - comparison of CK values one week prior to surgery to CK values on the day after surgery; EA - emergence agitation *Significant difference by the Kruskal-Wallis test (p=0.01) **Significant difference by the Steel-Dwass test (p<0.01) ***Significant difference by the Steel-Dwass test (p<0.01)

Figure 1 shows the ΔCK values for each EA group. The median ΔCK values in the EA1 to EA5 groups were 3 (quartile −19~39), 5 (−32~88), 99.5 (−18~190.5), 121 (29~219.5), and 144 (41~340.5), respectively, indicating a statistically significant difference overall. Statistically significant differences were also observed between the EA1 and EA4 groups and between the EA2 and EA4 groups. 

**Figure 2:**
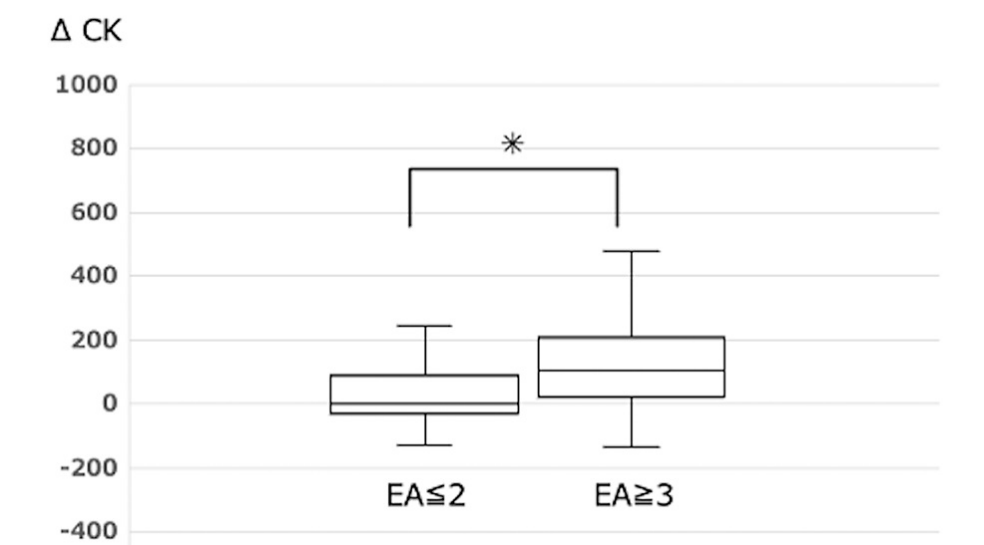
ΔCK in the EA ≤ 2 and EA ≥ 3 groups CK - creatine kinase; ΔCK - comparison of CK values one week prior to surgery to CK values on the day after surgery; EA - emergence agitation *Significant difference by the Mann-Whitney U test (p<0.01)

Figure 2 shows the ΔCK values for the EA ≤2 and EA ≥3 groups. The median ΔCK values in the EA ≤2 and EA ≥3 groups were 3 (quartile −27~85) and 108 (23.5~206.7), respectively, indicating a statistically significant difference.

**Figure 3:**
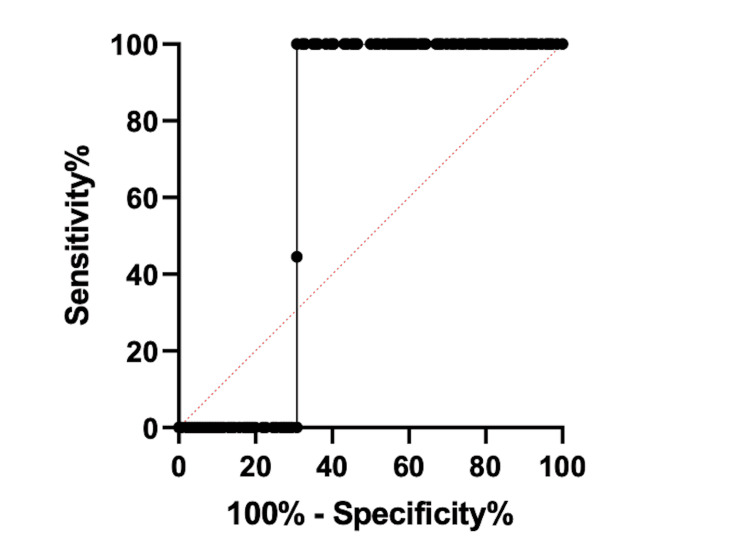
ΔCK level and emergence agitation CK - creatine kinase; EA - emergence agitation The respective AUC value of was 0.69 ± 0.038 (95% CI: 0.6169 to 0.7667, p<0.0001). The cut-off value of 2.00 U/L that was used to predict EA had 100% (95% CI: 97.4 to 100.0%) a specificity of 61.0% (95% CI: 61.3 to 76.1%) and a likelihood ratio of 3.24.

Figure 3 shows the ΔCK level and emergence agitation. The respective AUC value of was 0.69 ± 0.038 (95% CI: 0.6169 to 0.7667, p<0.0001). The cut-off value of 2.00 U/L that was used to predict EA had 100% (95% CI: 97.4 to 100.0%) a specificity of 61.0% (95% CI: 61.3 to 76.1%) and a likelihood ratio of 3.24.

## Discussion

In the past, we have noted several cases of high CK levels on the day after surgery among children who have undergone general anesthesia using volatile inhalation anesthetics. A detailed examination of a patient’s condition upon awakening from anesthesia revealed that the patient was in an excited state upon awakening, suggesting that the high CK level on the day after surgery was probably caused by muscle breakdown associated with vigorous movement. However, this was unpublished data, and the details were unknown. Based on the above background, we thought that preoperative CK might provide some clues when imagining situations in which patients are likely to be stimulated by drugs or other effects upon awakening from general anesthesia, and we focused on surgeries performed at our hospital on pediatric patients with cleft lip and palate. However, the number of cases in which the preoperative CK exceeded the upper limit of normal was very small, and the relationship between the preoperative CK exceeding the upper limit of normal and EA in children could not be examined. Therefore, in this study, we examined the amount of postoperative CK elevation change compared to the preoperative value (ΔCK).

The results of the present study revealed that a higher EA score at the time of anesthesia awakening was associated with a larger ΔCK, indicating that a high CK level on the day after surgery is highly related to the state of the patient upon awakening. Although CK elevation is informative for MH diagnosis [8], it was suggested that CK elevation in the awake state above EA3 is due to elevation associated with being abated, and therefore, if the patient is in a good general condition, follow-up can be performed.

Normal CK levels tend to be higher in younger patients, and age must be considered in the evaluation of abnormal values. When CK is divided by age, pre- and postoperative CK values tended to increase with decreasing age. When this is divided by EA group above trend was true in the EA4 group. However, in this study, the age of patients in the EA5 group was higher than that of patients in the EA4 group, suggesting that abnormal CK levels after anesthetic awakening may be independent of age. 

In this study, it was found that an increase in EA upon awakening was associated with a greater likelihood of an increase in CK postoperatively. Various medications, including benzodiazepines, ketamine, and propofol, were used to reduce the incidence of EA [10]. However, there is no well-established prophylaxis or treatment for EA. Although supplemental opioids and/or sedatives are often used to reduce the incidence and severity of EA, anesthesiologists should always consider the risk of postoperative respiratory complications. Dexmedetomidine (DEX), a potent α2-adrenoceptor agonist, has sedative, analgesic, and anxiolytic properties without respiratory depression [11]. Some studies have shown the effectiveness of DEX in postoperative recovery in a pediatric population undergoing tonsillectomy, adenoidectomy, and palatoplasty [12-14]. Recently, DEX is also increasingly used for procedural sedation during awake fiberoptic intubation [15] and colonoscopy [16]. If EA can be suppressed prophylactically by means such as administering DEX, it may be possible to suppress the rise in CK as well.

This study has several limitations. The first is that the surgical population is limited to cleft lip and palate-related patients. If the target patients change, the results may change. The second is the large variability among the groups. Further studies are needed in the future with a smaller variability in a variety of pediatric surgical patients.

## Conclusions

We prospectively examined the relationship between the awakening state and CK on the day after surgery in children who have undergone anesthesia with volatile inhalation anesthetics with consideration of age. The results revealed that a higher EA score at the time of anesthesia awakening was associated with a larger ΔCK, indicating that a high CK level on the day after surgery is highly related to the state of the patient upon awakening. However, this was found to be independent of age.
